# The burden of comorbidity in people with chronic kidney disease stage 3: a cohort study

**DOI:** 10.1186/s12882-015-0189-z

**Published:** 2015-12-01

**Authors:** Simon D. S. Fraser, Paul J. Roderick, Carl R. May, Natasha McIntyre, Christopher McIntyre, Richard J. Fluck, Adam Shardlow, Maarten W. Taal

**Affiliations:** Academic Unit of Primary Care and Population Sciences, Faculty of Medicine, University of Southampton, South Academic Block, Southampton General Hospital, Tremona Road, Southampton, Hampshire SO16 6YD UK; Faculty of Health Sciences, University of Southampton, Southampton, UK; The Department of Renal Medicine, Royal Derby Hospital NHS Foundation Trust, Derby, Derbyshire UK; Division of Medical Sciences and Graduate-Entry Medicine, University of Nottingham, Derby, UK

**Keywords:** Chronic kidney disease, Comorbidity, Multimorbidity, Polypharmacy, Mortality

## Abstract

**Background:**

Multimorbidity is a growing concern for healthcare systems, with many countries experiencing demographic transition to older population profiles. Chronic kidney disease (CKD) is common but often considered in isolation. The extent and prognostic significance of its comorbidities is not well understood. This study aimed to assess the extent and prognostic significance of 11 comorbidities in people with CKD stage 3.

**Methods:**

A prospective cohort of 1741 people with CKD stage 3 was recruited from primary care between August 2008 and March 2010. Participants underwent medical history, clinical assessment, blood and urine sampling. Comorbidity was defined by self-reported doctor-diagnosed condition, disease-specific medication or blood results (hemoglobin), and treatment burden as number of ongoing medications. Logistic regression was used to identify associations with greater treatment burden (taking >5 medications) and greater multimorbidity (3 or more comorbidities). Kaplan Meier plots and multivariate Cox proportional hazards models were used to investigate associations between multimorbidity and all-cause mortality.

**Results:**

One thousand seven hundred forty-one people were recruited, mean age 72.9 +/−9 years. Mean baseline eGFR was 52 ml/min/1.73 m^2^. Only 78/1741 (4 %) had no comorbidities, 453/1741 (26 %) had one, 508/1741 (29 %) had two and 702/1741 (40 %) had >2. Hypertension was common (88 %), 30 % had ‘painful condition’, 24 % anemia, 23 %, ischaemic heart disease, 17 % diabetes and 12 % thyroid disorders. Median medication use was 5 medications (interquartile range 3–8) and increased with degree of comorbidity. Greater treatment burden and multimorbidity were independently associated with age, smoking, increasing body mass index and decreasing eGFR. Treatment burden was also independently associated with lower education status. After median 3.6 years follow-up, 175/1741 (10 %) died. Greater multimorbidity was independently associated with mortality (hazard ratio 2.81 (95 % confidence intervals 1.72–4.58), *p* < 0.001) for 3 or more comorbidities vs 0 or 1).

**Conclusions:**

Isolated CKD was rare and multimorbidity the norm in this cohort of people with moderate CKD. Increasing multimorbidity was associated with greater medication burden and poorer survival. CKD management should include consideration of comorbidities.

## Background

Chronic kidney disease (CKD) is often viewed in isolation and clinical guidelines for the condition commonly focus on the consequences of impaired glomerular function and proteinuria [1, 2]. This approach neglects the reality that the prevalence of CKD rises steeply with age and it is therefore likely to occur in the setting of multiple comorbid conditions including hypertension, diabetes and cardiovascular disease [3]. ‘Multimorbidity’ is usually defined as having two or more chronic morbidities, therefore people with CKD who have one or more comorbidity meet this definition [3]. Comorbidities are important because they may impact on treatment burden, medications management, quality of life, and survival [4–6]. In addition multiple comorbidity and associated polypharmacy have major implications for patients’ capacity to cope with treatment as considered in a ‘burden of treatment model’ [7, 8]. This describes the actions that patients are required to undertake to successfully manage their condition as ‘work’ (both self-management and in interaction with health services) and their ability to respond appropriately as ‘capacity’ [7, 8]. As disease burden increases (due to disease complexity, severity or number of conditions) so does the work required of patients, and their capacity to respond may suffer, leading to poor outcomes as shown in other chronic conditions such as stroke [9].

Optimal clinical management of CKD would therefore benefit from better understanding of the nature, extent and prognostic implications of its common comorbidities in combination. This has been studied to some extent in dialysis populations and comorbidities have been included in risk scores to aid clinical decision making in transplant evaluation in the elderly, but less studied in earlier CKD [10, 11]. In many countries, including the UK, early CKD and its common comorbidities are principally managed in primary care [12]. This study therefore aimed to describe the extent, distribution and survival implications of eleven comorbidities, and the associated medication burden, in a cohort of people with CKD stage 3 in a primary care setting.

## Methods

### Participants

Participants were recruited from 32 general practitioner surgeries for the Renal Risk in Derby (RRID) study, a prospective cohort study of CKD stage 3 in a primary care setting. Detailed methods for the RRID study have been published elsewhere [13]. Eligible participants were 18 years or over, met the Kidney Disease Outcomes Quality Initiative (KDOQI) criteria for CKD stage 3 (current guidelines at the time of recruitment), able to attend their GP surgery and to give informed consent. People with previous transplant or terminal illness were excluded. Screening and baseline visits were combined due to the large proportion of elderly participants and logistical challenges of conducting study visits in multiple primary care centers. First study visits occurred from August 2008 to March 2010, questionnaire information was checked, anthropomorphic measurements taken, urinalysis performed, and blood specimens taken. All participants provided written informed consent. The study was approved by Nottingham Research Ethics Committee 1 and included on the National Institute for Health Research Clinical Research Portfolio (NIHR Study ID:6632).

### Definitions

The eleven comorbidities included in these analyses were hypertension, diabetes, ischaemic heart disease, heart failure, peripheral vascular disease, cerebrovascular disease, chronic respiratory disorder, depression, chronic painful condition, thyroid disorder and anaemia. These comorbidities were chosen for pragmatic reasons including ease of identification from patient report, medication history, and laboratory data, and because they represent a broad spectrum of chronic conditions prevalent among older people.

Participants were asked to list chronic medications on a questionnaire including details of any regular pain medication taken. These were confirmed verbally at study visits and further verified by examination of repeat prescriptions where possible.

Blood pressure was measured after a minimum of five minutes rest in the sitting position, using a validated oscillometric device, recommended by the British Hypertension Society (Digital Blood Pressure Monitor Model UA-767, A & D Instruments Ltd, Abingdon, UK). The same device was used for all readings. BP was calculated as the mean of three readings that differed by <10 %. Hypertension was defined as taking current antihypertensive medication, or systolic BP >140 mmHg or diastolic BP >90 mmHg at baseline. Diabetes was defined by self-report of having a previous clinical diagnosis in line with World Health Organisation criteria or being on medication for diabetes [14]. Ischemic heart disease was defined as participant-reported myocardial infarction or coronary revascularisation procedure. Heart failure was defined as patient reported clinical diagnosis. Peripheral vascular disease was defined as peripheral arterial revascularization or amputation. Cerebrovascular disease was defined as participant-reported stroke or transient ischemic attack. Chronic respiratory disorder was defined by chronic use of inhaled beta-2 adrenergic agonists (either short or long acting) and / or inhaled steroid. The details of all reported comorbidities were checked verbally with participants at study vists. Depression was defined as ongoing use of a selective serotonin reuptake inhibitor or serotonin–norepinephrine reuptake inhibitor. Thyroid disorders were defined by taking thyroxine or carbimazole. Anemia was defined according to Kidney Disease Improving Global Outcomes (KDIGO) guidelines as haemoglobin <13.0 g/dl (<130 g/l) in males and <12.0 g/dl (120 g/l) in females at baseline [1]. Painful condition was defined as ongoing regular analgesia use.

Smoking status was categorized as never smoked, ex-smoker, and current smoker. Socioeconomic status (SES) was defined by two methods. First, using the Indices of Multiple Deprivation score (IMD); a small area social deprivation score comprising a composite measure of seven domains (income, employment, health and disability, education skills and training, barriers to housing and other services, crime and living environment) [15]. Second, using self-reported education status; an important indicator of socioeconomic status in elderly populations [16]. Education status was categorized into three groups (1: no formal qualifications, 2 : school or equivalent qualifications, 3: degree or equivalent). Self-reported ethnicity information was collected and, due to the small number of non-white participants, categorized into ‘White’ and ‘Other’ for analysis. eGFR was calculated using the Chronic Kidney Disease Epidemiology Collaboration (CKDEPI) equation [17]. Albuminuria was defined as albumin/creatinine ratio (uACR) ≥3 mg/mmol in at least two of the three urine specimens. uACR was fitted as a continuous variable in regression analyses as log of the mean of three uACR values. Body mass index (BMI) was calculated from weight in kilograms divided by height squared in meters [18].

### Outcomes

Participants were registered with the Health and Social Care Information Centre to obtain date and cause of death. The observation period was from date of recruitment until 24th February 2013. Cause of death was as recorded on the death certificate. Causes of death were independently reviewed by three investigators and classified as cardiovascular, cancer, infection or other. Classification differences were resolved by discussion.

### Statistical analyses

Descriptive statistics were used to summarise the frequency and distribution of the numbers and types of comorbidities and the numbers of medications at baseline. These were described in terms of having isolated CKD, CKD plus one, two, or more than two comorbidities, and taking less than five, more than five, or more than ten medications. Chi square tests were used to compare categorical variables.

Univariate and multivariable logistic regression models were used to identify associations with greater treatment burden (defined as taking more than five medications) and greater multimorbidity (defined as more than two comorbidities). Specific conditions were not included in the treatment burden analysis as several conditions were defined by medication status. A Kaplan Meier plot for all-cause mortality and Cox proportional hazards models were used to describe survival by degree of comorbidity. Cox regression models were developed with multimorbidity fitted as a categorical variable (comparing people with more than two vs. two comorbidities vs none or one) with subsequent addition of sociodemographic (age, sex, education status, IMD) and then lifestyle and clinical variables (smoking, BMI, eGFR, uACR). The final model included variables with a p value < 0.10 on univariate analysis. Proportional hazards assumptions were checked using Nelson–Aalen plots. Interactions between age and smoking, sex and smoking and comorbidity and smoking were checked because of the potential for these factors to modify the mortality effect of smoking [19].

## Results

A total of 1741 people were recruited to the RRID study. The study population was predominantly white (>98 %) and elderly. Mean age was 72.9 +/−9 and 67 % were over 70 years. Mean baseline eGFR was 52 ± 10 ml/min/1.73 m^2^ (911 participants (52.3 %) were CKD stage 3a, 386 participants (22.2 %) were stage 3b). Isolated CKD was uncommon; only 78/1741 (4 %) had no comorbidities in the list considered, 453/1741 (26 %) had at least one comorbidity, 508/1741 (29 %) had two comorbidities of the list considered and 702/1741 (40 %) had more than two comorbidities. At baseline, having three or more comorbidities was more common in men, older people, people with CKD G3b, ex-smokers, and people with greater socioeconomic deprivation, lower educational attainment, higher BMI, or any albuminuria (Table 1). Hypertension was the commonest comorbidity and painful condition the second (Table 2). The median number of medications at baseline was five (interquartile range 3–8); 1033/1741 (59 %) were taking five or more medications and 198/1741 (11 %) ten or more and only 46 (3 %) were taking no medication. Greater comorbidity burden was associated with taking higher numbers of medications (*p* < 0.001 for trend, Table 1). On multivariable logistic regression, greater treatment burden (taking more than five medications) and greater multimorbidity (three or more comorbidities) were both independently associated with increasing age, smoking, increasing BMI and decreasing eGFR (Table 3). Greater treatment burden was also independently associated with lower education status (Table 3).

**Table 1:** Characteristics of people in the renal risk in derby cohort by number of comorbidities

		Number of comorbidities								
Characteristic	Category	0		1		2		3 or more		Total *n* = 1741
		Total *n* = 78		Total *n* = 453		Total *n* = 508		Total *n* = 702		
		n	(row %)	n	(row %)	n	(row %)	n	(row %)	(n (% of total) unless otherwise stated)
Sex*	Male	15	(2.2)	174	(25.3)	200	(29.0)	300	(43.5)	689 (39.6)
	Female	63	(6.0)	279	(26.5)	308	(29.3)	402	(38.2)	1052 (60.4)
Age*	<60	22	(17.2)	50	(39.1)	27	(21.1)	29	(22.7)	128 (7.4)
	60–69	26	(5.8)	146	(32.8)	121	(27.2)	152	(34.2)	445 (25.6)
	70–79	23	(3.0)	186	(24.4)	224	(29.4)	328	(43.1)	761 (43.7)
	80+	7	(1.72)	71	(17.4)	136	(33.4)	193	(43.4)	407 (23.4)
Ethnicity	White	78	(5.0)	443	(26.1)	497	(29.3)	680	(40.1)	1698 (97.5)
	Other	0	(0)	10	(23.3)	11	(25.6)	22	(51.2)	43 (2.5)
Index of multiple deprivation*	Quintile 1 (most deprived)	5	(3.3)	32	(21.2)	42	(27.8)	72	(47.7)	151 (8.7)
	Quintile 2	27	(6.3)	103	(23.8)	118	(27.3)	184	(42.6)	432 (24.8)
	Quintile 3	14	(4.3)	88	(27.0)	94	(28.8)	130	(39.9)	326 (18.7)
	Quintile 4	12	(2.7)	128	(28.6)	135	(30.2)	172	(38.5)	447 (25.7)
	Quintile 5 (least deprived)	20	(5.2)	100	(26.1)	118	(30.9)	144	(37.7)	382 (21.9)
Education status*	Group1 (No formal qualifications)	30	(3.2)	209	(21.9)	305	(32.0)	409	(42.9)	953 (54.7)
	Group 2 (GCSE, A level, NVQ1-3)	33	(7.0)	130	(27.7)	126	(26.9)	180	(38.4)	469 (26.9)
	Group 3 (1st or higher degree, NVQ 4–5)	15	(4.7)	113	(35.7)	77	(24.3)	112	(35.3)	317 (18.2)
eGFR at study entry*	Mean (SD)	57.8	(7.6)	55.8	(9.7)	51.7	(10.2)	50.3	(10.5)	52.5 (10.4)
	>60	30	(7.2)	163	(39.0)	107	(25.6)	118	(28.2)	418 (24.0)
	45–59	44	(4.8)	227	(24.9)	277	(30.4)	363	(39.9)	911 (52.3)
	30–44	4	(1.0)	61	(15.8)	113	(29.3)	208	(53.9)	386 (22.2)
	<30	0	(0)	2	(7.7)	11	(42.3)	13	(50.0)	26 (1.5)
Albuminuria - based on 2 of 3 samples*	No albuminuria	76	(5.2)	402	(27.4)	433	(29.5)	558	(38.0)	1469 (84.4)
	Albuminuria A2 ≥ 3 mg/mmol but <30 mg/mmol)	2	(0.9)	45	(19.4)	66	(28.5)	119	(51.3)	232 (13.3)
	Albuminuria A3 (≥30 mg/mmol)	0	(0)	6	(15.0)	9	(22.5)	25	(62.5)	40 (2.3)
Smoking*	Current	7	(8.6)	31	(38.3)	15	(18.5)	28	(34.5)	81 (4.7)
	Ex-smoker	30	(3.5)	169	(19.5)	264	(30.5)	403	(46.5)	866 (49.7)
	Never	41	(5.2)	253	(31.9)	229	(28.8)	271	(34.1)	794 (45.6)
BMI*	Normal or underweight	31	(8.8)	103	(29.2)	105	(29.8)	114	(32.2)	353 (20.3)
	Overweight	32	(4.3)	207	(28.1)	214	(29.0)	285	(38.6)	738 (42.4)
	Obese	15	(2.3)	143	(22.0)	189	(29.1)	303	(46.6)	650 (37.3)
Number of medications*	None	28	(60.9)	16	(34.8)	2	(4.4)	0	(0)	46 (2.6)
	1–2	37	(15.6)	128	(53.8)	61	(25.6)	12	(5.0)	238 (13.7)
	3–4	10	(2.4)	198	(46.7)	155	(36.6)	61	(14.4)	424 (24.4)
	5–9	3	(0.4)	109	(13.1)	269	(32.2)	454	(54.4)	835 (48.0)
	10 or more	0	(0)	2	(1.0)	21	(10.6)	175	(88.4)	198 (11.4)

Conditions included: diabetes, hypertension, ischaemic heart disease, heart failure, peripheral vascular disease, cerebrovascular disease, respiratory condition, depression, painful condition, thyroid disorder, anaemia

*Abbreviations*: *GCSE* General certificate of secondary education, *A level* advanced level, *NVQ* National vocational qualification, *BMI* Body mass index

*eGFR* estimated glomerular filtration rate, *SD* standard deviation

*p < 0.05 test for trend

**Table 2:** Prevalence of individual comorbidities at baseline in the RRID study

Comorbidity	n	Prevalence
Hypertension	1528	87.8 %
Painful condition	530	30.4 %
Anaemia	418	24.0 %
Ischaemic heart disease	398	22.9 %
Diabetes	294	16.9 %
Thyroid disorder	208	11.9 %
Cerebrovascular disease	200	11.5 %
Respiratory condition	181	10.4 %
Depression	94	5.4 %
Peripheral vascular disease	82	4.7 %
Heart failure	61	3.5 %

**Table 3:** Associations of greater treatment burden and greater multimorbidity

Variable		More than five medications per day				Three or more comorbidities			
		(vs. five or fewer)				(vs. two or fewer)			
		Univariate		Multivariable^a^		Univariate		Multivariable^a^	
		OR (95 % CI)	*p* value	OR (95 % CI)	*p* value	OR (95 % CI)	*p* value	OR (95 % CI)	*p* value
Age (years)		1.05 (1.03–1.06)	<0.001	1.04 (1.03–1.06)	<0.001	1.04 (1.02–1.05)	<0.001	1.03 (1.02–1.05)	<0.001
Sex (male vs. female)		1.26 (1.03–1.53)	0.02	1.14 (0.92–1.43)	0.231	1.25 (1.03–1.52)	0.027	1.09 (0.88–1.36)	0.434
Education status (vs. highest level of qualifications)	Group1 (No formal qualifications)	1.83 (1.41–2.36)	<0.001	1.44 (1.10–1.90)	0.01	1.38 (1.06–1.79)	0.035	1.14 (0.86–1.52)	0.361
	Group 2 (GCSE, A level, NVQ1-3)	1.29 (0.97–1.72)		1.27 (0.94–1.73)		1.14 (0.85–1.53)		1.13 (0.82–1.55)	
Smoking (vs. non smokers)	Current smoker	1.02 (0.64–1.61)	<0.001	1.49 (0.91–2.44)	<0.001	1.02 (0.63–1.65)	<0.001	1.38 (0.83–2.28)	<0.001
	Ex smoker	1.57 (1.29–1.92)		1.45 (1.18–1.79)		1.68 (1.38–2.05)		1.54 (1.25–1.91)	
BMI (kg/m^2^, continuous)		1.08 (1.05–1.10)	<0.001	1.09 (1.07–1.11)	<0.001	1.06 (1.04–1.08)	<0.001	1.07 (1.05–1.10)	<0.001
eGFR (ml/min per 1.73 m^2^, continuous)		0.96 (0.95–0.97)	<0.001	0.97 (0.96–0.98)	<0.001	0.97 (0.96–0.98)	<0.001	0.97 (0.96–0.98)	<0.001
Log average uACR (mg/mmol, continuous)		1.00 (1.00–1.00)	0.70	-	-	1.00 (0.99–1.00)	0.384	-	-

*p* values for variables with multiple categories represent p for trend

*Abbreviations*: *GCSE* General certificate of secondary education, *A level* advanced level, *NVQ* National vocational qualification, *BMI* Body mass index, *eGFR* estimated glomerular filtration rate, *uACR* urinary albumin to creatinine ratio, *SD* standard deviation

^a^Adjusted for age, sex, education status, smoking, BMI and eGFR

**Fig. 1:**
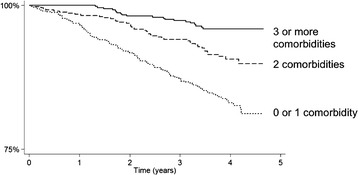
Kaplan Meier plot showing cumulative survival (all-cause mortality) by comorbidity status. Footnote to Fig. 1: Please note that the x axis does not cross the y axis at 0 %

Overall mean follow-up time was 3.6 ± 0.8 years (1317 ± 287 days). 175 participants (10 %) died during follow up and 1537 (90 %) remained alive. Those who died tended to be older, male, have fewer educational qualifications, have a history of smoking, and have CVD and/or diabetes. The commonest cause of death was CVD (41 %) followed by cancer (29 %). People with two or more comorbidities experienced poorer survival (Fig. 1). On univariate Cox regression analyses, male sex, age, history of smoking, lower BMI, lower eGFR, albuminuria, and greater number of comorbidities were associated with increased risk of all-cause mortality (full data not shown). After adjustment for socio-demographic variables, the relationship between number of comorbidities and all-cause mortality was attenuated (from 4.58 (95 % confidence intervals (CI) 2.85–7.38) to 3.15 (95 % I 1.95–5.10, *p* < 0.001) for three or more compared to one or no comorbidities). Greater number of comorbidities, increasing age, male sex, ex-smoking and lower eGFR remained significantly associated with increased risk of all-cause mortality in the final model (adjusting for age, sex, lifestyle and clinical variables, Table 4). No interactions were identified.

**Table 4:** Cox proportional hazards models for all-cause mortality in the RRID study population

Variable		Model 1^a^		Model 2^b^		Model 3^c^	
		(univariate)		(sociodemographic variables)		(lifestyle and clinical variables)	
		HR (95 % CI)	*p* value	HR (95 % CI)	*p* value	HR (95 % CI)	*p* value
Number of comorbidities (vs none or one)	2	2.31 (1.36–3.94)	<0.001	1.68 (0.99–2.88)	<0.001	1.51 (0.88–2.59)	<0.001
	3 or more	4.58 (2.85–7.38)		3.15 (1.95–5.10)		2.81 (1.72–4.58)	
Age (years)		-	-	1.08 (1.06–1.11)	<0.001	1.07 (1.05–1.09)	<0.001
Sex (male vs. female)		-	-	1.74 (1.28–2.36)	<0.001	1.45 (1.06–1.97)	0.020
Education status (vs. highest level of qualifications)	Group1	-	-	1.60 (0.96–2.68)	0.112	-	-
	(No formal qualifications)						
	Group 2	-	-	1.45 (0.92–2.32)		-	-
	(GCSE, A level, NVQ1-3)						
Index of multiple deprivation (vs. least deprived)	Quintile 1 (most deprived)	-	-	1.40 (0.90–2.19)	0.900	-	-
	Quintile 2	-	-	1.36 (0.85–2.22)		-	-
	Quintile 3	-	-	1.35 (0.85–2.13)		-	-
	Quintile 4	-	-	1.23 (0.66–2.27)		-	-
Smoking (vs. never smokers)	Current smoker	-	-	-	-	1.20 (0.86–1.66)	0.049
	Ex-smoker	-		-		2.07 (1.01–4.25)	
BMI (kg/m^2^, continuous)		-	-	-	-	0.98 (0.95–1.02)	0.319
eGFR (ml/min per 1.73 m^2^, continuous)		-	-	-	-	0.96 (0.95–0.98)	<0.001
Log average uACR (mg/mmol, continuous)		-	-	-	-	0.99 (0.99–1.00)	0.885

Outcome – all cause mortality at Feb 2013, n events = 175

*Abbreviations*: *BMI* Body mass index, *eGFR* estimated glomerular filtration rate, *uACR* urinary albumin to creatinine ratio, *SD* standard deviation

^a^Univariate

^b^Adjusted for age, sex, education status, IMD

^c^Final model adjusted for age, sex, smoking, BMI, eGFR and uACR

## Discussion

In this cohort study of predominantly older people with mild to moderate CKD we found high levels of comorbidity and polypharmacy and demonstrated that increased comorbidity was associated with reduced survival. While our list of included comorbidities was not exhaustive, our findings demonstrate that, even in a cohort recruited in primary care, CKD rarely occurs in isolation. Virtually all patients were ‘multimorbid’ according to the usual definition of having two or more chronic morbidities [3]. Our finding that only 4 % of people with CKD stage 3 had no comorbidity is striking and clinically important.

### Strengths and limitations

This study had several strengths, including large numbers of people with CKD, being conducted in a primary care setting, ascertainment of a broad range of comorbidities by interview rather than routine data, and prospective follow-up. However, we recognise important limitations. We did not have an age-matched control group without CKD with whom to compare multimorbidity burden. It is also likely that we under-identified some comorbidities by use of patient self-report (such as heart failure) and medication-definition; depression and respiratory disorders, for example, were defined by medication only, thus reducing their prevalence in our study. We were unable to include certain important CKD comorbidities such as cancer and liver disease because people with terminal illness were excluded and, although participants were asked about co-exisiting conditions, specific questions about all forms of cancer were not included in the baseline questionnaire. In addition, the prevalence of patient-reported liver disease was considered too low to meaningfully include in analyses and thought likely to be under-ascertained by patient self-report as largely asymptomatic (data not shown). The use of a medication-driven definition will, to some extent, be a reflection of the ‘work’ patients are required to do in managing a condition, but defining conditions by medication meant that we were unable to include them as variables in our logistic regression models. This reduced our ability to examine whether medication burden had greatest association with specific conditions. There is also no agreed method of defining morbidities. We considered causative factors and potential CKD complications if they affected quality life per se or treatment burden through their management, and or prognosis. Anaemia and hypertension were included for these reasons. We did not include obesity as this is largely asymptomatic, though we recognise that following lifestyle advice maybe burdensome and symptoms may develop as obesity increases. Including obesity as an additional comorbidity would have increased overall burden. These uncertainties would benefit from further discussion and consensus in people with CKD [20].

We were unable to consider frailty (which has been shown to be associated with CKD) or cognitive impairment both of which may influence patient capacity and outcome, or other outcomes such as quality of life (which might be more sensitive to some of our morbidities than mortality), this will be assessed in later follow up stages of this cohort [21–23]. Similarly, our assessment of comorbidities does not account for disease severity. Conditions causing pain, such as osteoarthritis, were not identified individually. Participants volunteered for this study, so we may have selected a population with *less* comorbidity and lower frailty. We also had no data on the burden of health care (such as clinic visits) or medication adherence. The combined effects of these limitations is that even the high prevalence of comorbidities that we have reported is likely to be an underestimate.

### Comparison with existing literature

The debate regarding the relevance of early or mild CKD has often focussed on the hypothetical person with reduced GFR and no other medical problems but our data show that this combination is only present in a very small minority [24]. In the majority, CKD was associated with other medical problems and 40 % had more than two comorbidities. Multiple studies have shown that the presence of CKD increases the risk of adverse outcomes associated with a wide range of other diseases and, as we have shown, greater comorbidity is associated with increased all-cause mortality even in mild to moderate CKD [25–28]. In a historical prospective cohort study of people with CKD identified from electronic patient records, Gullion et al. identified that, compared to age and sex matched controls without CKD, people with CKD had higher levels of comorbidity and higher risk of mortality for the same degree of comorbidity [29]. Thus there is mutual amplification of risks associated with CKD and comorbid conditions. Taken together these observations imply that it is important to consider the implications of CKD for the integrated care of patients with multimorbidity. The presence of multiple comorbid conditions has important implications for medicines management [30]. We identified very high prevalence of polypharmacy with 59 % of people taking five or more medications and 11 % taking ten or more. Furthermore, perhaps unsurprisingly, a greater number of comorbidities was associated with a greater number of medications. We showed that taking more than five medications was independently associated with older age and lower education status. Many commonly used medications require dose adjustments for reduced GFR and polypharmacy is associated with increased risk of adverse drug interactions and risk of acute kidney injury [31]. As the majority of patients with CKD are elderly and more susceptible to adverse drug effects, our data suggest that careful medicines management (including coordination between pharmacists and community and hospital physicians) should form an important part of the care of people with CKD.

Burden of treatment is a relatively under-studied consideration in CKD, the more common focus being burden of illness [32–34]. The cumulative complexity of developing new comorbidities and the balance of work and capacity have implications for the success of self- and shared-management efforts, increasingly recognised in other chronic conditions such as stroke and heart failure [9, 35, 36]. The burden of comorbidities is usually higher in groups with lower socioeconomic status and/or lower educational attainment as we have shown in this cohort [3]. Lower socioeconomic status is also linked to lower health literacy - a key component of patients’ capacity [37]. An unexpected but important finding of our study was the high prevalence of certain conditions in this population, for example ‘painful condition’ and depression, though both were probably underestimated in our study due to reliance on medication for their definition. Both may also adversely influence capacity and are common in older people in general population studies in the UK [38, 39]. Thus our data confirm that mild CKD is associated with high disease burden, high treatment burden and reduced capacity to cope with the demands of treatment. These factors should be considered when developing and agreeing care plans for people with CKD.

### Implications for research and practice

A patient-centred approach to managing patients with multimorbidity (such as that recommended by the American Geriatrics Society) is in line with evidence that risks of fragmentation of care and medical error related to multimorbidity can be ameliorated by a dedicated clinician acting in an overseeing role [40–42]. Generalists in integrated primary care teams are usually best placed to offer this continuity of care and clinical oversight in mild to moderate CKD though, as CKD advances, nephrologists commonly adopt this role. Improving care coordination has the potential to improve outcomes and reduce health care costs for people with multimorbidity. This includes improving medicines management as discussed above. Informed decision-making is challenging in multimorbidity and there is need to be judicious in adding to patients’ treatment burden, particularly for treatments that do not relieve symptoms but reduce future risk. This may be particularly important in older patients with CKD, whom we have shown have a high prevalence of comorbidities and reduced capacity.

We were unable to describe severity of the comorbid conditions or quality of life in this study, which represent important considerations for future research. There are also implications of the high prevalence of comorbidities to clinical trial design in people with CKD (where people with comorbidities may be excluded from trials). Further research should also investigate to what extent comorbidities may be caused or exacerbated by CKD (e.g. anaemia).

## Conclusions

In this cohort of people with moderate CKD, we identified that isolated CKD was rare and multimorbidity the norm. Polypharmacy as a measure of treatment burden was common, linked to degree of comorbidity and associated with older age and lower education status. Survival was independently associated with greater number of comorbidities. Integrated care for people with CKD should go beyond a focus on reduced GFR or albuminuria and include consideration of the burden of comorbidities (and their treatments) balanced against patient capacity to cope with further investigation and treatment.

### Meetings

This (or similar data from this study) was presented as a poster at the American Society of Nephrology Kidney Week Conference, Philadelphia, November 2014.

It was also presented at the British Society of Nephrology conference, Leeds, UK July 2015 and as a poster at the European Renal Association congress, London, UK, May 2015.

## Abbreviations

CKD: Chronic kidney disease
RRID: Renal Risk in Derby (study)
NHS: National Health Service
uACR: Urinary albumin to creatinine ratio
eGFR: Estimated glomerular filtration rate
AKI: Acute kidney injury
RAASi: Renin-angiotensin aldosterone system inhibitors
CVD: Cardiovascular disease
NICE: National Institute for Health and Care Excellence
IMD: Index of Multiple Deprivation
GP: General practitioner
BP: Blood pressure
SES: Socioeconomic status
KDOQI: Kidney Disease Outcomes Quality Initiative
KDIGO: Kidney Disease Improving Global Outcomes
