# Correction to: The burden of comorbidity in people with chronic kidney disease stage 3: a cohort study

**DOI:** 10.1186/s12882-020-02205-w

**Published:** 2020-12-21

**Authors:** Simon D. S. Fraser, Paul J. Roderick, Carl R. May, Natasha McIntyre, Christopher McIntyre, Richard J. Fluck, Adam Shardlow, Maarten W. Taal

**Affiliations:** 1 Academic Unit of Primary Care and Population Sciences, Faculty of Medicine, University of Southampton, South Academic Block, Southampton General Hospital, Tremona Road, Southampton, Hampshire SO16 6YD UK; 2 Faculty of Health Sciences, University of Southampton, Southampton, UK; 3 The Department of Renal Medicine, Royal Derby Hospital NHS Foundation Trust, Derby, Derbyshire UK; 4 Division of Medical Sciences and Graduate-Entry Medicine, University of Nottingham, Derby, UK

**Correction to: BMC Nephrol 16, 193 (2015)**

**https://doi.org/10.1186/s12882-015-0189-z**

Following publication of the original article [1], the authors identified an error in Fig. 1. The labels for ‘3 or more comorbidities’ and ‘0 or 1 comorbidity’ were incorrectly placed and should be reversed on this Kaplan Meier plot (i.e. people with 3 or more comorbidities experienced poorer survival and people with 0 or 1 comorbidity experienced better survival). The correct figure is given below.

**Fig. 1:**
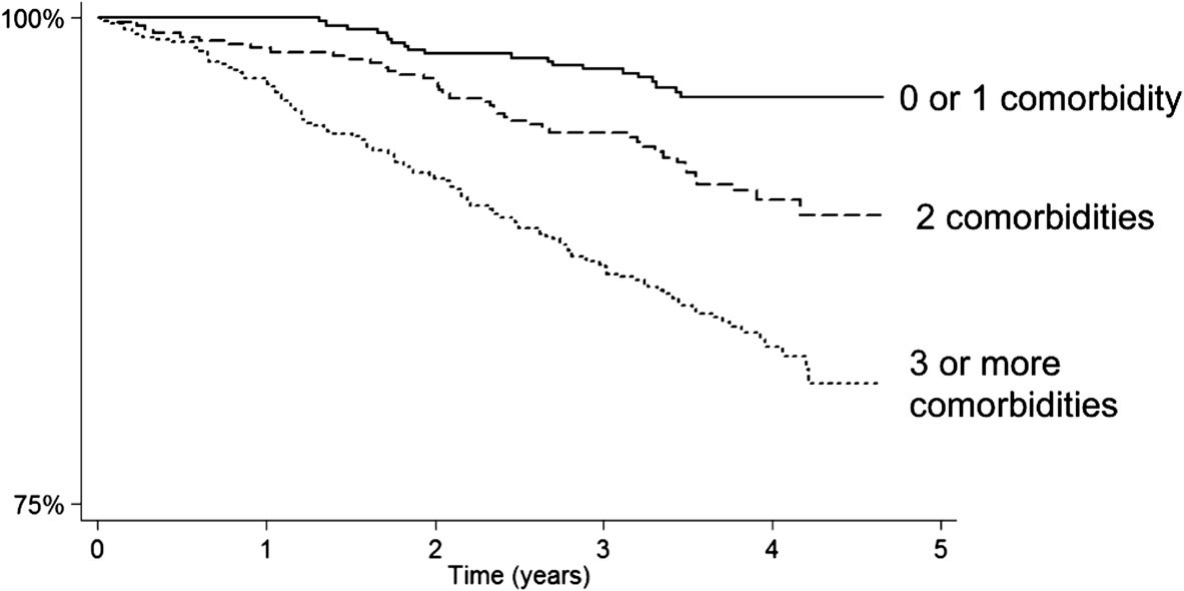
Kaplan Meier plot showing cumulative survival (all-cause mortality) by comorbidity status. Footnote to Fig. 1: Please note that the x axis does not cross the y axis at 0 %
